# Targeted treatment of severe vascular malformations harboring *PIK3CA* and *TEK* mutations with alpelisib is highly effective with limited toxicity

**DOI:** 10.1038/s41598-023-37468-4

**Published:** 2023-06-28

**Authors:** Martin Sterba, Petra Pokorna, Renata Faberova, Blanka Pinkova, Jarmila Skotakova, Anna Seehofnerova, Jan Blatny, Lucia Janigova, Olga Koskova, Hana Palova, Michal Mahdal, Lukas Pazourek, Petr Jabandziev, Ondrej Slaby, Peter Mudry, Jaroslav Sterba

**Affiliations:** 1 Department of Paediatrics, Faculty of Medicine, University Hospital Brno, Masaryk University, Brno, Czech Republic; 2 Department of Biology, Faculty of Medicine, Central European Institute of Technology, Masaryk University, Brno, Czech Republic; 3 Department of Paediatric Oncology, Faculty of Medicine, University Hospital Brno, Masaryk University, Cernopolni 212/9, 613 00 Brno, Czech Republic; 4 Department of Paediatric Radiology, Faculty of Medicine, University Hospital Brno, Masaryk University, Brno, Czech Republic; 5 Department of Paediatric Surgery, Orthopaedics and Traumatology, Faculty of Medicine, University Hospital Brno, Masaryk University, Brno, Czech Republic; 6 1st Department of Orthopaedics, Faculty of Medicine, St. Anne’s University Hospital Brno, Masaryk University, Brno, Czech Republic; 7 International Clinical Research Center, St. Anne’s University Hospital, Brno, Czech Republic

**Keywords:** Medical research, Molecular medicine

## Abstract

This was a prospective cohort study of eighteen patients with large and debilitating vascular malformations with one or more major systemic complications. In all patients, we discovered activating alterations in either *TEK* or *PIK3CA*. Based on these findings, targeted treatment using the PI3K inhibitor alpelisib was started with regular check-ups, therapy duration varied from 6 to 31 months. In all patients, marked improvement in quality of life was observed. We observed radiological improvement in fourteen patients (two of them being on combination with either propranolol or sirolimus), stable disease in 2 patients. For 2 patients, an MRI scan was not available as they were shortly on treatment, however, a clinically visible response in size reduction or structure regression, together with pain relief was observed. In patients with elevated D-dimer levels before alpelisib administration, a major improvement was noted, suggesting its biomarker role. We observed overall very good tolerance of the treatment, documenting a single patient with grade 3 hyperglycemia. Patients with size reduction were offered local therapies wherever possible. Our report presents a promising approach for the treatment of VMs harboring different targetable *TEK* and *PIK3CA* gene mutations with a low toxicity profile and high efficacy.

## Introduction

Vascular anomalies (VA) are a large and diverse group of diseases characterized by abnormal growth and development of blood or lymphatic vessels. They are associated with diverse symptomatology and often disabling conditions and remain both diagnostic and therapeutic challenges for medical professionals all over the world^1,2^. According to the ISSVA 2018 classification (Classification of Vascular Anomalies ©2018 International Society for the Study of Vascular Anomalies Available at “issva.org/classification” Accessed 01-JUN-2023), they comprise two major categories, vascular tumors and vascular malformations, which can be further divided into several subgroups. Vascular malformations (VMs) can be categorized into simple, combined, vascular malformations of major named vessels and vascular malformations associated with other anomalies.

The overall incidence of congenital VMs in the general population is 1.5%, and approximately two-thirds of cases are of venous predominance^3^. They can present as localized or diffuse lesions, and the symptomatology depends on the localization, extension, and involved anatomical structures. The appearance and symptoms are not static and can often progress during growth spurts and puberty^1,2^. Because of this varied symptomatology, multidisciplinary cooperation is of vital importance. For extensive lesions with vital organ and body part involvement, surgical procedures are very complicated because of their size and/or location. In these patients, other therapeutic options should be considered.

Mutations in genes that are involved in two significant intracellular signaling pathways, RAS/MAPK and PI3K/AKT, have been implicated in the pathophysiology of the majority of vascular malformations^4–6^. This opens the possibility that drugs/inhibitors currently being used in cancer patients may be used to treat patients with VMs^7^.

In this prospective observation report, we focused on the effect of the PI3K inhibitor alpelisib, which was recently approved by the FDA and EMA for the treatment of *PIK3CA*-mutated, hormone receptor-negative advanced breast cancer, showing very promising results^8^. Several authors have already demonstrated its effect in patients with *PIK3CA*-related overgrowth syndromes or *PIK3CA*-altered lymphatic malformations, and as a result of the EPIK-P1 clinical study^9^, it has recently been approved by the FDA for adult and pediatric patients with severe manifestations of the *PIK3CA*-related overgrowth spectrum^10–13^. In addition to patients with activating *PIK3CA* mutations, alpelisib treatment might also be beneficial for patients with activating *TEK* alterations, as the PI3K/AKT pathway is considered to be a central part of signaling through the TIE2 receptor encoded by this gene^14^. This was recently demonstrated in work published by Remy et al., in which the authors described the efficacy and pharmacokinetics of alpelisib in 3 patients with VMs harboring *TEK* mutation^15^.

## Results

In each patient, activating mutations in either *TEK* or *PIK3CA* were found. In 12/18 patients, *TEK* exon 17 mutation was found, with *TEK* p.L914F being the most prevalent (9/12). In one of these patients, we found a *TEK* mutation of germline origin (p.Y897C). One patient harbored a truncating mutation in exon 23 of the *TEK* gene. Five patients harbored *PIK3CA* hotspot mutations, all of which have already been observed in patients with VM. In one patient, in addition to the somatic *TEK* mutation found in VA biopsy, a germline *PTPN11* mutation causing Noonan syndrome was detected. The identified variants and the testing methods for each patient are listed in Table 1.

**Table 1:** List of patients with basic characteristics and clinical symptoms, class of VM, results of molecular analysis, and sequencing methods used.

Patient	Gender	Age (y)	Gene alteration	Method	Class of VM according to ISSVA classification and clinical manifestation
1	F	9	*TEK* c.2740C > T/p.L914F	WES	VeM of left lower limb, buttocks, and pelvis, intraosseous infiltration, movement limitations, pain
2	F	19	*TEK* c.2740C > T/p.L914F	WES	VeM of right lower limb, buttocks, pelvis, and genitals, chronic DIC, bleeding, immobility, pain
3	F	18	*TEK* c.2740C > T/p.L914F, *PTPN11* c.598A > T/p.N200Y (germline)	WES	VeM of the left scapula and upper limb, chronic DIC, movement limitation, pain
4	F	11	*PIK3CA* c.1258 T > C/p.C420R	WES	VeM of the left chest and abdominal wall, scoliosis, pain
5	F	14	*TEK* c.2740C > T/p.L914F	Sanger sequencing	VeM of right cheek, pain, headaches
6	F	3	*TEK* c.2690A > G/p.Y897C (germline)	Sanger sequencing	Multiple VLM of the cranium, subcutaneously on the head, thorax, and limbs
7	M	19	*PIK3CA* c.1633G > A/p.E545K	NGS	Sporadic AVM of left lower limb involving m. semimembranosus, movement limitation, pain
8	F	21	*PIK3CA* c.3140A > G/p.H1047R	NGS	VeM of the right foot, pain, inability to walk
9	M	17	*TEK* c.2740C > T/p.L914F	Sanger sequencing	VeM of right lower limb, genitals, movement limitations, pain, coagulopathy
10	F	17	*TEK* c.2753G > A/p.R918H	Sanger sequencing	VeM of right upper limb and shoulder, chronic coagulopathy, pain, DIC
11	F	37	*TEK* c.2752C > T/p.R918C	WES	VeM of left lower limb and pelvis, movement limitations, pain
12	F	22	*PIK3CA* c.1633G > A/p.E545K	NGS	VeM/hemangioma of left lower limb involving m. vast. intermed., inoperable, pain, movement limitations
13	M	9	*TEK* c.2740C > T/p.L914F	NGS	VeM of right lower limb (involving m. gluteus max., m. semitendinosus, m. vastus intermedius, m. semimembranosus, m. biceps femoris), inoperable, pain, movement limitations, chronic coagulopathy
14	F	14	*PIK3CA* c.3140A > G/p.H1047R	NGS	VeM of left lower lim assoc. with Klippel-Trenaunay syndrome, chronic coagulopathy
15	F	3	*TEK* c.3323_3324del/p.Y1108*	WES	VeM of the face forming from right ear to chin involving muscles of oral cavity and tongue
16	F	7	*TEK* c.2740C > T/p.L914F	Sanger sequencing	VeM of the left knee, intramuscular involvement, pain, movement limitations
17	F	2	*TEK* c.2740C > T/p.L914F	NGS	Generalized VeM of the neck, oro- and nasopharynx, back, chest wall, mediastinum, buttocks, thighs, knees, and left shin and foot
18	M	11	*TEK* c.2740C > T/p.L914F	Sanger sequencing	VeM of the right hand, pain, mobility impairment

*VM* vascular malformation, *VeM* venous malformation, *VLM* venolymphatic malformation, *AVM* arteriovenous malformation, *M* male, *F* female.

The total duration of alpelisib treatment varied from 6 to 31 months as of May 2023.

In all patients, marked improvement in the quality of life (QoL) was observed. In 12 patients, radiological improvement was documented; in 2 patients, the anomaly size remained stable; in 2 patients, radiological improvement was documented after combining alpelisib with either sirolimus (No. 6) or propranolol (No. 9). For 2 patients (Nos. 16 and 17), an MRI scan was not available due to short period of alpelisib administration. However, rapid clinical response with subjective improvement in QoL was documented. Effect of the treatment for every single patient is summarized in Table 2, percentage of VM size reduction in pre- and posttherapy MRI scans was radiologically measured either as volumatic, or planar change. For this purpose, we excluded two patients on combined treatment and two patients without MRI at time of analysis.

**Table 2:** List of patients with the treatment indication, duration of treatment, starting dose and the effect of the treatment on quality of life (QoL), size on MRI, and coagulation markers.

Patient	Age at treatment initiation (years)	Indication for treatment^a^ (P / M / C)	Duration of treatment^b^ and initial dose	QoL	MRI (% of volume reduction comparing pre- and posttherapy imaging) (* Planar size reduction)	D-dimer level	Side effect	Compliance
1	7	P, M, C	24 months (150 mg/day)	Marked improvement of pain, lesion tenderness, and mobility after 1 month of the treatment	Stable	Marked improvement after 3 months of the treatment	None	Well tolerated
2	17	P, M, C	30 months (200 mg/day)	Marked improvement of pain, lesion tenderness, and mobility after 1 month of the treatment	*41% reduction after 6 months of the treatment	Marked improvement after 3 months of the treatment, elevation when treatment paused	IDDM, headache, menorrhagia, abdominal pain	Need to temporary pause the treatment due to IDDM
3	16	P, M, C	31 months (200 mg/day)	Marked improvement of pain, lesion tenderness, and mobility after 6 weeks of the treatment	20% reduction after 2 months of the treatment	Marked improvement after 6 months of the treatment, elevation when treatment paused	Headaches when combined with trametinib	Well tolerated as a single agent
4	8	P, M	30 months (200 mg/day)	Marked improvement of pain after 3 months of the treatment	41% reduction regression after 6 m of the treatment	Within normal range before the start of the treatment	Hypertension	Well tolerated
5	12	P, M, C	24 months (300 mg/day)	Marked improvement of pain and lesion tenderness after 3 months of the treatment	34% reduction after 8 months of the treatment	Marked improvement after 2 months of the treatment	Hair loss, nausea	Well tolerated in reduced dose
6	1	P, M, C	23 months (80 mg/day)	Marked improvement of pain and lesion tenderness after 3 months of the treatment	Progression of lesions after 6 months of the treatment, 23% reduction after 4 months of alpe/sirolimus combination	Marked improvement after 1 month of the treatment	None	Well tolerated
7	17	P, M	23 months (200 mg/day)	Marked improvement of pain and mobility after 1 month of the treatment, recurrent pain after one month of alpelisib pause	30% reduction after 6 months of the treatment	Within normal range before the start of the treatment	None	Well tolerated
8	19	P, M	Stopped after 9 months on patient request (200 mg/day)	Marked improvement of pain and mobility after 1 month of the treatment	61% reduction after 6 months of the treatment	Within normal range before the start of the treatment	Hyperglycemia on oral hypoglycemic agents, gastritis	Initially well tolerated, discontinued based on patient request
9	15	P, M, C	22 months (300 mg/day)	Marked improvement of pain and mobility after 1 month of the treatment	Stable after 6 months of the treatment, 25% reduction after 4 months of alpe/propranolol combination	Improvement after 6 months of the treatment	None	Well tolerated
10	15	P, M, C	18 months (300 mg/day)	Marked improvement of pain, lesion tenderness, and mobility after 1 month of the treatment	57% reduction after 5 months of the treatment, stationary/slight progression after 12 months of the treatment	Marked improvement after 1 month of the treatment	Hyperglycemia without the need for a specific medication	Well tolerated
11	35	P, M, C	30 months (300 mg/day)	Marked improvement of pain, lesion tenderness, and mobility after 1 month of the treatment	*11% reduction after 16 months of the treatment	Marked improvement after 3 months of the treatment	None	Well tolerated
12	20	P, M	Stopped after 6 months due to resection of the lesion (200 mg/day)	Marked pain reduction and mobility improvement	87% reduction after 3 months of the treatment, lesion operable	Within normal range before the start of the treatment	Headaches	Well tolerated
13	8	P, M, C	9 months (200 mg/day)	Marked improvement of pain, lesion tenderness, and mobility after 1 month of the treatment	Stable	Marked improvement after 1 month o the treatment	None	Well tolerated
14	13	P, M, C	14 months (200 mg/day)	Marked improvement of pain, lesion tenderness, and mobility after 3 months of the treatment	*45% reduction after 4 months of the treatment	Marked improvement after 1 month of the treatment	None	Well tolerated
15	2	P, C	12 months (100 mg/day)	Lesions discolored and less tender after 2 months of the treatment	19% reduction after 6 months of the treatment	Marked improvement after 3 months of the therapy	None	Well tolerated
16	6	P, M, C	10 months (150 mg/day)	Marked improvement of pain, lesion tenderness, and mobility after 1 month of the treatment	N/A at the time of publication	Marked improvement after 3 months of the treatment	None	Well tolerated
17	2	P, M, C	6 months (40 mg/day)	Improvement of pain, lesions discolorized, less tender after 3 months of the treatment	N/A at the time of publication	Marked improvement after 3 months of the treatment	None	Well tolerated
18	11	P, M, C	6 month (200 mg/day)	Marked improvement of pain and mobility after 3 months	5% reduction after 3 moonths of the treatment	Within normal range before the start of the treatment	None	Well tolerated

^a^Indications for the treatment: *P* pain, *M* mobility impairment, *C* Coagulopathy.

^b^Duration of treatment as of May 2023: *QoL* quality of life, *IDDM* insulin dependent diabetes mellitus, *N/A* not applicable.

When comparing patients with *TEK* (9 patients) and *PIK3CA* (5 patients) mutation, better radiological response was observed in *PIK3CA* mutation group. For *TEK* mutation group the average volume reduction was 21% and median value was 19%. For *PIK3CA* mutation group the average volume reduction was 53% and median value was 45%. Moreover, both patients with stable disease harbored *TEK* mutation.

**Figure 1:**
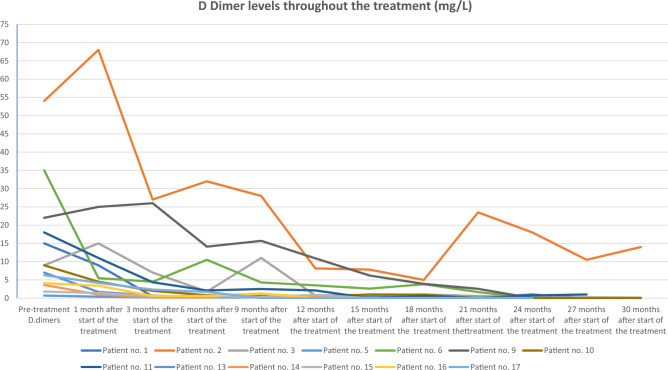
D-dimer levels throughout alpelisib treatment in patients whose levels were outside the normal range before treatment initiation.

In patients with elevated D-dimer levels before alpelisib administration, a major improvement was noted. The D-dimer levels continually decreased, as shown in Fig. 1.

Notable individual cases are further described in more detail below.

We observed overall very good tolerance of the treatment. According to the CTCAE v5.0^16^, we noted grade 3 toxicity of hyperglycemia requiring insulin treatment in one patient and grade 2 toxicity in 4 patients. Those specifically included mucositis in two patients, hyperglycemia requiring oral hypoglycemic medication and recurrent abdominal pain in one. The rest of the reported side effects were only grade 1, most commonly temporary abdominal discomfort, headaches, nausea, hyperglycemia, or liver enzyme elevation. Apart from the patient with hyperglycemia on insulin treatment, none of the patients required admission for treatment of adverse events, and all were managed in an outpatient setting.

### Index cases presentation

**Figure 2:**
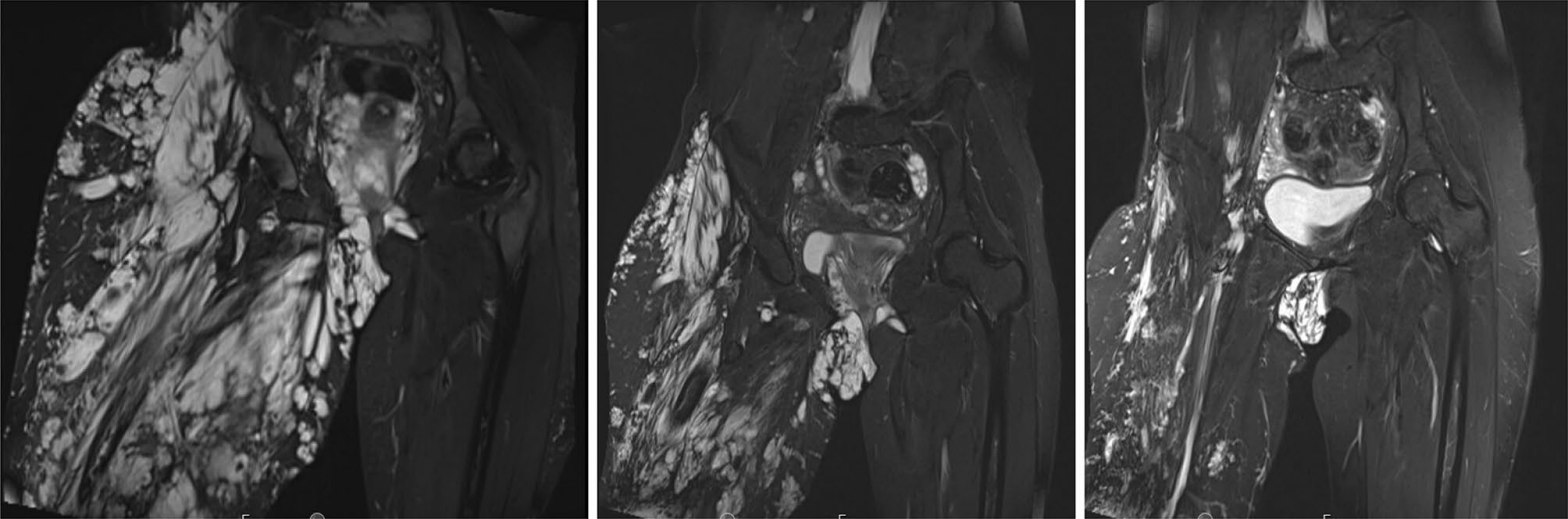
Patient number 2. MRI (T2 STIR cor. plane) of the pelvis and proximal thighs prior to (left), 6 months middle) and 12 months after alpelisib treatment (right).

One of the first patients (No. 2) who was administered alpelisib treatment was an 18-year-old woman with severe and debilitating venous malformation of the right lower limb, pelvis, and genitalia. Prior to the start of alpelisib in November 2020, she underwent several surgical procedures over the years with only temporary effects. After 5 months of alpelisib treatment, we observed a rapid size reduction (see Fig. 2) and significant improvements in both QoL measurements and coagulation markers.

**Figure 3:**
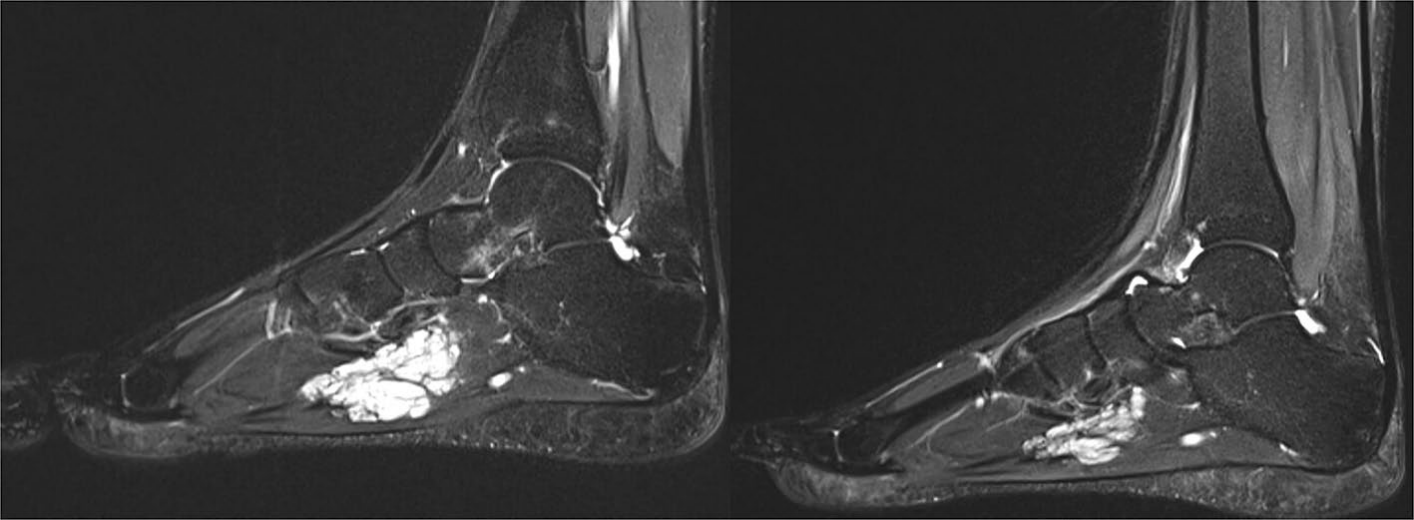
Patient number 8. MRI (T2 STIR sagit. plane) of the right foot prior (left) and 3 months after alpelisib treatment (right).

Patient No. 8 came to our clinic with a VeM located in her right foot, which resulted in severe pain while walking. Within weeks after the introduction of alpelisib treatment, she was able to walk and soon even run without any limitations. The effect of the treatment on the VeM size reduction and structure regression is well documented on MRI of the right foot prior to and 3 months after alpelisib treatment (see Fig. 3).

The patient with Noonan syndrome (No. 3) and a somatic *TEK* mutation had a rapid but only partial effect of the single-agent alpelisib treatment; therefore, a MEK inhibitor was added to target *PTPN11* germline mutation, which may have contributed to the pathogenesis and thus to enhance the effect of alpelisib. However, very soon after the start of this double-agent therapy, the patient suffered from severe headaches and nausea, leading to the temporary discontinuation of both drugs. Within 2 weeks of discontinuation, we observed a rapid increase in the size of the malformation and D-dimer levels. Therefore, alpelisib treatment was restarted as monotherapy at a 50% dose reduction with good patient compliance and both clinical and laboratory efficacy in decreasing D-dimer levels.

The oldest patient was a 36-year-old woman who underwent a series of 25 surgeries and multiple nontargeted treatments for her VeM of a left lower limb starting in childhood before genetic testing discovered a pathogenic mutation of the *TEK* gene. After starting alpelisib treatment, we observed rapid clinical effects in QoL and size reduction of the lesions, which was later confirmed with MRI imaging.

**Figure 4:**
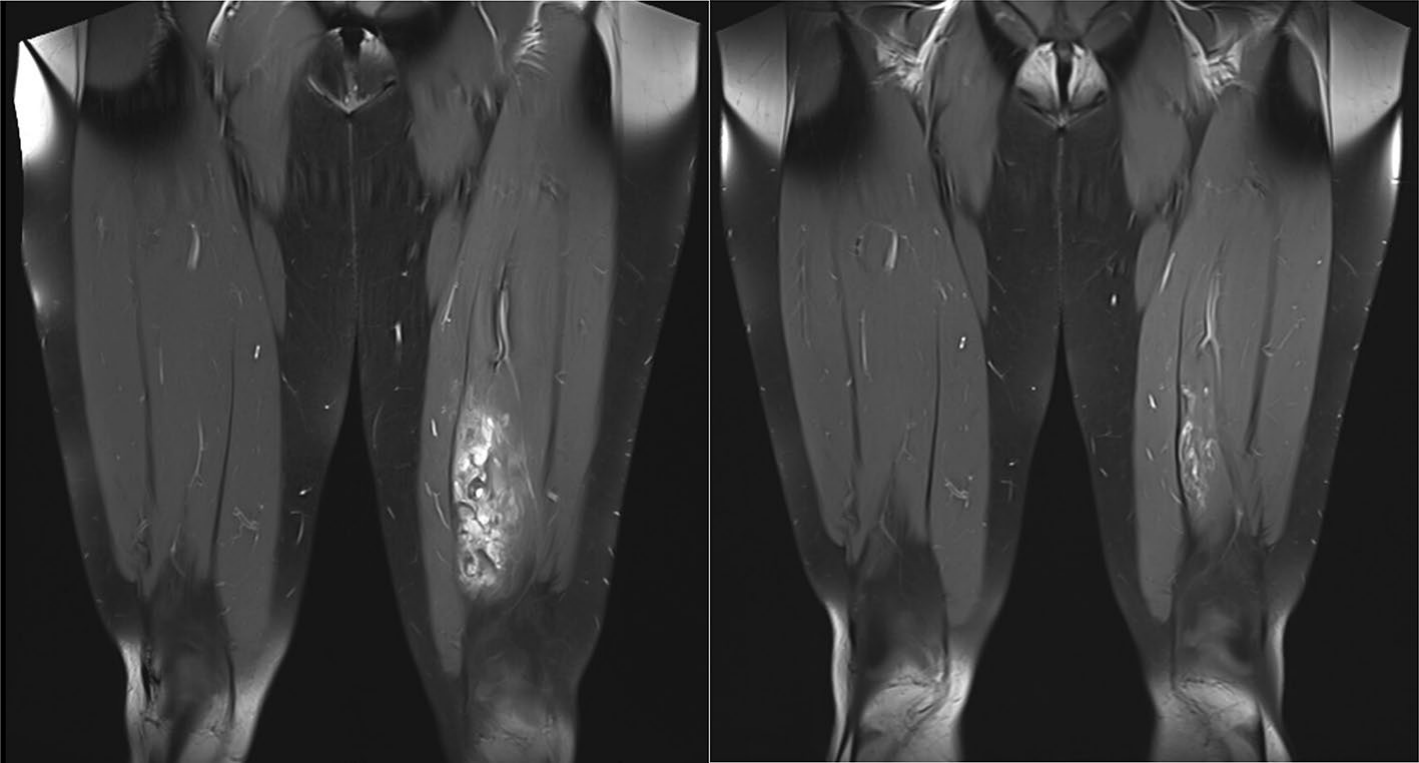
Patient number 12. MRI (T1 STIR cor. Plane) of the thighs prior (left) and 3 months after alpelisib treatment (right).

Patient no. 12, with VeM of the left lower limb with muscular involvement, started the treatment due to potentially disabling surgery. Six months after the alpelisib administration, we achieved size reduction allowing safe total resection of the lesion without collateral damage to the healthy tissue. Six months after the procedure, the patient remains asymptomatic without any treatment (See Fig. 4).

## Discussion

The use of targeted therapeutics for the management of VMs represents a new off-label therapeutic strategy, which could be used as an addition to classic local treatment approaches following the paradigm of precision medicine. Drugs that are currently being used in this setting include mTOR inhibitors^17^, PI3K inhibitors^11^, AKT inhibitors^18^, MEK inhibitors^19^, and VEGF inhibitors^7^; however, some of them show only limited efficacy, possibly because they are administered without prior knowledge of the driving gene mutation.

In this observational study, activating mutations in the *PIK3CA* and *TEK* genes were discovered in the lesions of 18 patients. Molecular analyses were performed using various sequencing methods ranging from Sanger sequencing to next-generation sequencing of targeted gene panels or WES. These different methods were selected based on current availability and the clinical picture suggesting a particular mutation in a given patient. The best diagnostic algorithm for the future, reflecting both economic and medical needs, remains to be established and is the subject of a future project.

Obtaining a representative piece of the lesion for subsequent analyses can become the first major obstacle due to the high risk of severe bleeding. A multidisciplinary team of specialists is of vital importance in facing such challenges. In patients with a difficult surgical approach, liquid biopsy could potentially help to discover driving mutations and may serve as a potential diagnostic marker; however, further investigation is needed^20^.

The starting dose of alpelisib was selected based on BSA-modified dosing schemes for the treatment of patients with breast cancer for whom a single dose of 300 mg is recommended (calculated 173 mg/m^2^ BSA). Our study used a 20% dose reduction (138.6 mg/m^2^/day) with high efficacy. Thus, for patients with insufficient response, the dose can be increased. In addition, for one of our patients who suffered an adverse event, restarting the drug with a 50% reduction was sufficient to maintain efficacy with a response measurable clinically and in D-dimer levels. In patient with hyperglycemia grade 3 toxicity we observed regrowth of the lesion after alpelisib cessation, restarting drug in 25% of the original dose led to suboptimal clinical and D-dimer response, however, at 37% of the original dose there was a very good both clinical and laboratory effect with only low dose of insulin therapy. Management of alpelisib induced diabetes mellitus was recently described in case report by Pia Peris et al.^21^.

To evaluate the therapeutic effect, patients underwent regular check-ups consisting of current history taking, clinical investigation, and laboratory testing. With the more recent patients, we also performed MRI scans before and 6 months after the start of the treatment. A measurable response was observed in 14 out of 18 patients on MRI, and a visual clinical response was observed in all patients, proving that alpelisib is highly effective. In all patients, we observed a striking effect on clinical symptomatology within the first few months of the treatment. Similar efficacy was reported by other authors describing their experience in a cohort of 19 patients and two case reports of patients with *PIK3CA*-related overgrowth syndrome (PROS)^11–13^. In our study, we also report the efficacy of alpelisib on VMs in patients without germline mutations but with somatic mutations, and moreover, this report describes alpelisib efficacy in the largest cohort of patients with somatic mutations in the *TEK* gene so far, thus following up on work of Remy et al.^15^.

When compared the radiological response in patients with *TEK* and *PIK3CA* mutations, both the average volume reduction and median value were higher in patients harboring *PIK3CA* mutation, however larger group would be needed for stronger results. The response in QoL parameters and coagulation markers were similar for both groups.

In patients who originally suffered from chronic coagulopathy, alpelisib treatment resulted in a decrease and even normalization of D-dimer levels, which seems to be a very useful marker of treatment success. Furthermore, we observed pain relief with quality-of-life improvement as the most important therapeutic goal. We have not observed any signs of cumulative toxicity comparing the first and second 6 months of drug exposure thus far.

After treatment discontinuation in two patients, we observed both clinical and paraclinical deterioration with a rapid rise of D-dimer levels, suggesting the need for prolonged treatment exposure. However, the optimal treatment duration and minimal effective dose remain unknown, as does the long-term toxicity profile in young patients. Different dosing and scheduling schemes have yet to be tested. Another open question is identifying the so-called “best achievable response” and reintroducing different local therapeutic options. Subjects who achieve size reduction after alpelisib treatment can be recommended for local treatments with a much lower risk of mutilation or complications such as bleeding or anatomical structure destruction. In our cohort, one patient (No. 12) experienced a 87% volume reduction of initially unresectable lesions on alpelisib, and the residual lesions were resected without bleeding or functional deficits of the thigh.

This prospective observational study contributes informations about a new treatment option that is well tolerated and shows objective responses with highly appreciated quality of life improvement.

## Methods

All patients or their legal guardians signed informed consent with molecular genetic testing and off-label treatment using alpelisib. A total of 18 patients with large, debilitating or opioid requiring VMs with one or more major systemic complications (e.g., chronic systemic consumption coagulopathy, major organ involvement or multiple bone and joint involvement, scoliosis) underwent molecular genetic analysis using either whole-exome sequencing (WES), a targeted next-generation sequencing (NGS) panel, or direct sequencing of *TEK* exon 17 by Sanger’s method. The analyses were performed on lesion biopsy specimens. DNA was extracted from FFPE tissue using QIAmp FFPE Tissue Kit (Qiagen, Germany) and treated with NEBNext FFPE DNA Repair Mix (New England Biolabs, MA, USA). For WES, both lesion and matched normal genomic DNA were used. Libraries were prepared using TruSeq DNA Exome (Illumina, CA, USA) according to the manufacturer’s instructions and sequenced on the NextSeq500 platform using NextSeq 500/550 Mid Output Kit v2.5 (150 Cycles) (Illumina). Libraries for targeted NGS were prepared using QIAseq Targeted DNA Panel—Human Actionable Solid Tumor (Qiagen) according to the manufacturer’s instructions and sequenced on the NextSeq500 platform using NextSeq 500/550 Mid Output Kit v2.5 (300 Cycles) (Illumina). For direct sequencing of exon 17 of the *TEK* gene, custom-made PCR primers (IDT, NJ, USA) were used to amplify the target sequence (F: 5′-CCTGGGTGGTGTTGCTAGAT-3′, R: 5′-AGAGGGAACTCCACAGGAAAG-3′). Sequencing analysis of a purified and labeled PCR product was performed on the ABI 3130xl device (ThermoFisher Scientific, MA, USA). Patients with confirmed mutations in *PIK3CA* and *TEK* were recruited for alpelisib treatment and efficacy analysis.

All patients included in our study were Caucasian. Prior to the start of alpelisib treatment, a thorough patient history, clinical examination, baseline imaging, and laboratory tests, including D-dimer levels, were obtained. Initial clinical symptoms with basic demographic data, class of VM according to ISSVA (International society for the study of vascular anomalies) and discovered mutation types are summarized in Table 1.

Based on molecular findings and clinical symptoms, all 18 patients began orally administered alpelisib treatment. The dosage used for our patients was derived from the breast cancer guidelines and individually adjusted to body surface area (BSA) and varied from 50 to 300 mg per dose. The average starting dose was 138.6 mg/m^2^/day. As our center was not part of the EPIK-P1 study, the dosing of alpelisib in our patients differs from the dosing schedule in EPIK-P1, which led to the FDA approval of alpelisib for *PIK3CA*-related Overgrowth Spectrum patients in April 2022^22^.

Regular check-ups were performed to evaluate the efficacy of the treatment. Each patient was examined in an outpatient clinic with laboratory and clinical examinations every 4 weeks and imaging, mostly MRI scan, every 3 months ± 2 weeks.

Quality of life (QoL) was assessed with a revised patient control outcome questionnaire which included an assessment of both pain and functional impairment resulting in a four-step semiquantitative scale: worse, stable, improved, and marked improvement.

Adverse events were classified according to CTCAE v5.0.

Discontinuation of the alpelisib treatment was allowed at the patient´s request due to adverse events and at the discretion of the treating physician.

All methods were carried out in accordance with relevant guidelines and regulations.

## Conclusion

Novel PI3K inhibitors such as alpelisib represent a promising therapeutic option for patients with confirmed *TEK* or *PIK3CA* gene alterations when local treatment methods have only partial or temporary effect or when the lesion is inoperable because of its location. This prospective observational study contributes information about this new treatment option, which is well tolerated documenting only 1 grade 3 toxicity, objective responses, and highly appreciated quality of life improvements.

Several questions still need to be answered, as this is an off-label, agnostic, and experimental treatment. Although we did not observe any side effect requiring permanent treatment discontinuation, long-term toxicity remains to be established. The optimal treatment duration, dosing, and scheduling are also a matter of further investigation, as well as the optimal position of this systemic treatment in the complex management of vascular malformations.

## Data Availability

The data that support the findings of this study are available from the corresponding author, [P. Mudry], upon reasonable request. Sequencing data are available from: https://www.ebi.ac.uk/ena/browser/view/PRJEB53413, accession number PRJEB53413.

## Abbreviations

BSA: Body surface area
CTCAE v5.0: Common terminology criteria for adverse events version 5.0
NGS: Next-generation sequencing
PIK3CA: Phosphatidylinositol-4,5-bisphosphate 3-kinase catalytic subunit alpha
PROS: PIK3CA-related overgrowth syndrome
QoL: Quality of life
TEK: Tyrosine kinase, endothelial
VA: Vascular anomalies
VM(s): Vascular malformation(s)
VeM: Venous malformation
WES: Whole-exome sequencing
